# Food Habits, Lifestyle Factors and Mortality among Oldest Old Chinese: The Chinese Longitudinal Healthy Longevity Survey (CLHLS)

**DOI:** 10.3390/nu7095353

**Published:** 2015-09-09

**Authors:** Zumin Shi, Tuohong Zhang, Julie Byles, Sean Martin, Jodie C. Avery, Anne W. Taylor

**Affiliations:** 1 School of Medicine, the University of Adelaide, Level 7 SAHMRI, North Terrace, Adelaide SA 5000, Australia; E-Mails: sean.martin@adelaide.edu.au (S.M.); jodie.avery@adelaide.edu.au (J.C.A.); anne.taylor@adelaide.edu.au (A.W.T.);; 2 School of Public Health, Peking University Health Science Center, 38 Xueyuan Road, Beijing 100191, China; E-Mail: tuohongzhang@126.com; 3 Priority Research Centre for Gender, Health and Ageing, School of Medicine and Public Health, Hunter Medical Research Institute, University of Newcastle, Newcastle NSW 2305, Australia; E-Mail: julie.byles@newcastle.edu.au

**Keywords:** diet, lifestyle factors, mortality, elderly, longitudinal study, Chinese

## Abstract

There are few studies reporting the association between lifestyle and mortality among the oldest old in developing countries. We examined the association between food habits, lifestyle factors and all-cause mortality in the oldest old (≥80 years) using data from the Chinese Longitudinal Healthy Longevity Survey (CLHLS). In 1998/99, 8959 participants aged 80 years and older took part in the baseline survey. Follow-up surveys were conducted every two to three years until 2011. Food habits were assessed using an in-person interview. Deaths were ascertained from family members during follow-up. Cox and Laplace regression were used to assess the association between food habits, lifestyle factors and mortality risk. There were 6626 deaths during 31,926 person-years of follow-up. Type of staple food (rice or wheat) was not associated with mortality. Daily fruit and vegetable intake was inversely associated with a higher mortality risk (hazard ratios (HRs): 0.85 (95% CI (confidence interval) 0.77–0.92), and 0.74 (0.66–0.83) for daily intake of fruit and vegetables, respectively). There was a positive association between intake of salt-preserved vegetables and mortality risk (consumers had about 10% increase of HR for mortality). Fruit and vegetable consumption were inversely, while intake of salt-preserved vegetables positively, associated with mortality risk among the oldest old. Undertaking physical activity is beneficial for the prevention of premature death.

## 1. Introduction

Living a long and healthy life is the ultimate goal for humans. Finding the secrets to longevity is a topic that has fascinated the scientist and non-scientist alike throughout the course of history. With advances in medicine and technology over recent decades, life expectancy has increased dramatically. Population ageing is now a global phenomenon especially in developed countries [1,2]. For example, in 2013 the estimated proportion of people aged 60 years or older was 32% in Japan and 14% in China [3]. In China, there were 23 million people aged 80 years and over in 2013 [4].

The determinants of longevity are not fully understood. It is commonly believed that multiple aetiologies (e.g., biological, environmental, and psychosocial factors) act together [5,6,7]. Lifestyle factors, such as physical activity, smoking, and food habits, play an important role [8]. However, much of the evidence is based on studies among populations in developed countries and with few people in the oldest age groups [9,10,11,12,13]. Studies on the oldest old often have had small samples and short follow-up duration [10,11,12,14] and often suffer from selective healthier survivor biases.

Diet is one of the major determinates of health. The beneficial effects of fruit and vegetable intake are well known including a reduction in the risk of mortality [15]. However, whether these effects still exist in very old people is less studied. The association between the intake of staple food (e.g., rice and wheat) and health outcomes remains understudied in the general population including the very old. While high intake of rice increases the risk of diabetes [16], it does not increase the risk of cardiovascular mortality and all-cause mortality in the general population [17,18]. A low carbohydrate and high protein diet has been advocated for weight loss. However, such a diet has been found to be associated with increased risk of mortality [19]. Previous studies have not specifically assessed the association between staple foods and mortality among the elderly [20].

The Chinese diet is characterized by a high intake of plant food and carbohydrate but low animal protein intake [21]. More than 80% of Chinese people aged 60 years and older have an intake of protein below the Chinese Dietary Reference Intake [22]. However, recent large epidemiological studies have found that a high intake of protein is associated with increased risk of mortality in the general population [19,23,24]. Whether a high intake of protein-rich food is protective against mortality among older Chinese is unknown.

Limited studies have assessed the association between food habits, lifestyle factors and mortality among the oldest old [25]. Using data from a large cohort of participants aged above 80 years, we aimed to study these associations.

## 2. Methods

### 2.1. Study Population

The study used data from the Chinese Longitudinal Healthy Longevity Survey (CLHLS). A detailed description of the study population has been previously published [26,27]. Briefly, in 1998 the baseline survey was conducted in 631 randomly selected counties and cities of 22 of China’s 31 provinces. Participants were followed up in 2000, 2002, 2005, 2008, 2009 and 2011. In total, 9093 participants participated in the 1998, 1999 baseline survey. In the analysis, we excluded 134 participants aged below 80 years, leaving 8959 participants for the current analysis. In total, 2206 (24.6%, 1169 urban, 1037 rural) participants were lost to follow-up during the study. The mean follow-up duration was 4.3 (S.D. ±2.9) years among those lost to follow-up. Table S1 shows the characteristics of those lost to follow-up. Those lost to follow-up were in general younger and more likely lived in an urban area.

This study was conducted according to the guidelines laid down in the Declaration of Helsinki, and all procedures involving human subjects/patients were approved by Biomedical Ethics Committee of Peking University (IRB00001052-13074). All participants signed a consent form.

### 2.2. Data Collection and Measurements

Participants were interviewed in their homes by health workers using a standard questionnaire (available online: http://centerforaging.duke.edu/documentation). All the interviewers were intensively trained before the survey.

#### 2.2.1. Dietary Measurements

Self-reported information on food consumption was collected through face-to-face interviews by trained research staff. The participants were asked to report their food frequency intake (both current and at the age of 60) of fruit, vegetable, meat, fish, beans, tea, garlic, egg and salt-preserved vegetables. Most of the frequencies were recorded as “almost every day” or “occasionally” or “rarely or never”. The frequency of fruit and vegetable intake was recorded as “almost every day”, “almost every day except in winter”, “occasionally” or “rarely or never”. In the analysis, we treated “almost every day except in winter” as almost every day.

Staple food pattern was assessed by the question “Please tell us the staple food you eat: (1) Rice, (2) Corn (maize), (3) Wheat (noodles and bread, *etc.*), (4) Other”. Participants were also asked for the amount of staple food intake in *liang* per day (*liang* is a Chinese unit, equals to 50 g).

#### 2.2.2. Healthy Lifestyle Score

A healthy lifestyle score was constructed based on three factors including daily intake of fruit, vegetable, and regular physical activity. A positive answer to each of the three lifestyle factors was given a score of 1. The maximum total score is 3.

#### 2.2.3. Death Ascertainment

In each follow-up survey, information on deaths and indicators of pre-death health status were collected through interviews with a close family member.

#### 2.2.4. Covariates

Cigarette smoking status was categorized into non-smokers (representing never smokers), ex-smokers and current smokers based on current and past history of smoking. The participants were asked whether they drink alcohol (yes/no) as well as the type and amount of alcohol they consumed. Information on regular physical activity was collected using question “Do you do exercise regularly at present, including jogging, playing ball, running and Qigong?” and recoded as yes or no. Participants were asked about whether they undertook a list of other eight activities including housework, growing vegetables/other field work, gardening, reading newspapers/books, raising domestic animals, playing cards and/or mah-jong, watching TV and or/listening to radio, and undertaking religious activities. A score was given to each activity based on weekly frequency: Almost every day (7), sometimes (3), never (0). A summary score of all these eight activities was created (range from 0 to 56) and recoded into quartiles. Education was grouped into four duration categories: 0 years, 1–5 years, 6–9 years, >9 years. Occupation before age of 60 was recoded into manual or non-manual based on a question with nine occupational categories. Participants were asked with whom they were living. The total number of chronic diseases was calculated as the sum of 13 conditions including hypertension, diabetes, heart disease, cardiovascular disease, bronchitis/emphysema/pneumonia/asthma, tuberculosis, cataracts, glaucoma, cancer, prostate tumour, gastric/duodenal ulcer, Parkinson’s disease and bedsores. The Katz Activities of Daily Living (ADL) Scale was used to assess participants’ disability [28]. Having difficulty performing any one or more of the ADL tasks (bathing, dressing, toileting, transfers, continence and eating) defined having an ADL disability.

### 2.3. Statistical Analysis

The Chi square test was used to compare differences in categorical variables and ANOVA in continuous variables. For each participant, person-years of follow-up were calculated from the date of the baseline survey to the date of death, lost to follow-up or the date of last follow-up in 2011, whichever came first. The association between food habits and lifestyle and all-cause mortality was analysed using Cox proportional hazard models, adjusting for multiple covariates. We provide both crude and adjusted hazard ratios (HRs). Two models assessed the association between food frequency intake and mortality. The first model controlled for age (continuous) and gender; the second model further adjusted for socio-demographic and lifestyle factors, job status before the age of 60, and residence (urban/rural). As the sample size in the full model was above 8300 (92.6% of the whole sample), we did not impute the missing data. In the sensitivity analyses we excluded those who died within the first year of the baseline survey or those having ADL disability. The proportional hazards assumption in the Cox model was assessed with graphical methods and with models including time-by-covariate interactions. In general, most of the proportionality assumptions were appropriate except meat intake, physical activity and other activities. Results from the Cox regression model with time-varying covariate were provided in addition to the conventional Cox regression.

We also used Laplace regression to model the association between food habits and lifestyle factors and median age at death (the age at which half of the participants had died and the other half were still alive), adjusting for other social demographic variables [29,30]. Results were put alongside the findings from the Cox models for easy comparison and interpretation.

Tests for interactions between food intake and sex were conducted by adding a multiplicative term between sex and the food frequency intake in the fully adjusted models. Because there was no significant gender by food intake interaction, we only present combined results. Statistical significance was considered when *p* < 0.05 (two-sided). All analyses were performed using Stata 13 (Stata Corp., College Station, TX, USA).

## 3. Results

Table 1 shows the sample characteristics at baseline. The mean age of the participants was 90.1 (standard deviation (S.D). ± 6.9) years in men and 93.8 (S.D. ± 7.7) years in women. Around 60% of the participants lived in rural areas. Half of the participants had rice as their staple food. The mean daily intake of staple food was 6.8 (S.D. ± 2.6) *liang* (1 *liang* = 50 g) in men and 5.7 (S.D. ± 2.3) *liang* in women. The mean number of chronic diseases was 0.9 (S.D. ± 1.1) in men and 0.8 (S.D. ± 0.9) in women. The participants were in general not overweight. The mean weight was 52.8 (S.D. ± 9.9) kg in men and 42.0 (S.D. ± 8.5) kg in women. The prevalence of alcohol drinking was 33.0% in men and 17.3% in women. Among alcohol drinkers, 56.6%, 34.1%, 8.6% reported drinking spirits, rice wine and wine, respectively. The mean alcohol intake (unconverted raw amount) was 2.5 *liang* per day (S.D. ± 2.4) among alcohol drinkers.

**Table 1:** Sample characteristics (*n* = 8959).

	Men	Women	*p*-value
*N*	3567	5392	
Age (years), mean (S.D.^1^)	90.1 (6.9)	93.8 (7.7)	<0.001
Weight (kg), mean (S.D.)	52.8(9.9)	42.0(8.5)	<0.001
Years of education			<0.001
0	1325 (37.1%)	4677 (86.7%)	
1–5	1413 (39.6%)	468 (8.7%)	
6–9	456 (12.8%)	112 (2.1%)	
>9	363 (10.2%)	95 (1.8%)	
Missing	10 (0.3%)	40 (0.7%)	
Co-residence			0.140
With household member(s)	3056 (85.7%)	4574 (84.8%)	
Alone	330 (9.3%)	569 (10.6%)	
Nursing home	181 (5.1%)	248 (4.6%)	
Missing	0 (0.0%)	1 (<1%)	
Residence			<0.001
Urban (city and town)	1426 (40.0%)	1952 (36.2%)	
Rural	2141 (60.0%)	3440 (63.8%)	
Smoking			<0.001
Current smoker	1116 (31.3%)	397 (7.4%)	
Ex-smoker	928 (26.0%)	445 (8.3%)	
Non-smoker	1520 (42.6%)	4547 (84.4%)	
Alcohol drink or not at present?			<0.001
Yes	1175 (33.0%)	930 (17.3%)	
No	2385 (67.0%)	4459 (82.7%)	
Exercise or not at present?			<0.001
No physical activity	2195 (61.6%)	4349 (80.8%)	
Having physical activity	1370 (38.4%)	1036 (19.2%)	
Quartiles of other activities score			<0.001
Q1 (0)	693 (19.4%)	1742 (32.3%)	
Q2 (3–7)	951 (26.7%)	1723 (32.0%)	
Q3 (9–14)	888 (24.9%)	1068 (19.8%)	
Q4 (15–56)	1035 (29.0%)	859 (15.9%)	
ADL disability			<0.001
No	4071 (60.3%)	1528 (69.3%)	
Yes	2658 (39.4%)	664 (30.1%)	
Missing	24 (0.4%)	14 (0.6%)	
Number of chronic diseases, mean (S.D.)	0.9 (1.1)	0.8 (0.9)	<0.001
Intake of fruit			0.045
Never	1037 (29.1%)	1548 (28.7%)	
Occasionally	1875 (52.7%)	2957 (54.9%)	
Almost daily	648 (18.2%)	883 (16.4%)	
Intake of vegetable			0.009
Never	161 (4.5%)	265 (4.9%)	
Occasionally	592 (16.6%)	1024 (19.0%)	
Almost daily	2807 (78.8%)	4100 (76.1%)	
Intake of meat			<0.001
Never	600 (16.9%)	1144 (21.4%)	
Occasionally	1731 (48.8%)	2717 (50.8%)	
Almost daily	1218 (34.3%)	1485 (27.8%)	
Intake of fish			<0.001
Never	1010 (28.5%)	1807 (34.0%)	
Occasionally	2055 (58.0%)	2860 (53.8%)	
Almost daily	476 (13.4%)	653 (12.3%)	
Intake of tea			<0.001
Never	1606 (46.8%)	3253 (63.3%)	
Occasionally	647 (18.9%)	850 (16.5%)	
Almost daily	1176 (34.3%)	1040 (20.2%)	
Intake of sugar			0.093
Never	1159 (32.8%)	1634 (30.6%)	
Occasionally	1459 (41.2%)	2288 (42.8%)	
Almost daily	920 (26.0%)	1421 (26.6%)	
Intake of salt-preserved vegetable			0.880
Never	1589 (45.1%)	2381 (44.8%)	
Occasionally	1161 (32.9%)	1775 (33.4%)	
Almost daily	775 (22.0%)	1153 (21.7%)	
Intake of garlic			<0.001
Never	1578 (45.1%)	2745 (52.3%)	
Occasionally	1443 (41.3%)	2028 (38.7%)	
Almost daily	476 (13.6%)	471 (9.0%)	
Intake of egg			<0.001
Never	650 (18.3%)	1154 (21.6%)	
Occasionally	1765 (49.7%)	2665 (49.9%)	
Almost daily	1138 (32.0%)	1520 (28.5%)	
Intake of bean			<0.001
Never	597 (16.8%)	1081 (20.2%)	
Occasionally	2024 (57.0%)	3130 (58.4%)	
Almost daily	932 (26.2%)	1151 (21.5%)	
Staple food intake (*liang*/day), mean (S.D.)	6.8 (2.6)	5.7 (2.3)	<0.001

^1^ S.D.: Standard Deviation.

There were 6626 deaths during 13 years of follow-up with 31,926 person-years. At the end of follow-up, the mean age was 97.6 (S.D. ± 5.6) years for survivors and 96.5 (S.D. ± 7.0) years for non-survivors. Each additional chronic disease was associated with 6% (95% CI (confidence interval) 3%–9%) increased risk of mortality.

Staple food patterns were not associated with mortality (Table 2). Compared with wheat as the staple food, the HRfor mortality of rice as the staple food was 1.01 (0.95–1.08). When we limited our analysis to those with rice as the staple food, there was an inverse association between rice intake and mortality in the multivariable model (adjusted for the same covariates as the multivariable model in Table 2) with HR of 0.98 (95% CI 0.97–0.99) for every 50 g of rice intake. No association between wheat intake and mortality was found among those with wheat as the staple food.

In the fully adjusted model, undertaking physical activity was associated with a 27% (95% CI 23%–32%) lower risk of mortality as compared with no physical activity (Table 3). Among all the lifestyle factors, physical activity had the highest effect on the differences of median age at death (1.18 years 95% CI 0.95–1.41). Women had about 20% lower risk of mortality than men. Having a manual job before the age of 60 years was associated with 37% increased risk of mortality as compared with those with a non-manual job. Participating in other activities was inversely associated with all-cause mortality (Table S2).

**Table 2:** Staple food patterns and mortality among Chinese elderly.

	*N*	HR (95% CI ^1^)		Differences (95% CI) in Median Age at Death (Years)	
		Unadjusted	Multivariable Model ^2^	Unadjusted	Multivariable Model ^2^
Staple food patterns					
Wheat	1708	1.00	1.00	Ref.	Ref.
Rice	6736	1.05 (0.99–1.12)	1.01 (0.95–1.08)	−0.24 (−0.44–−0.03) *	−0.12 (−0.32–0.08)
Maize	463	1.04 (0.93–1.18)	0.93 (0.83–1.05)	−0.39 (−0.76–−0.02) *	−0.19 (−0.51–0.13)
Others	45	1.02 (0.72–1.46)	1.04 (0.72–1.51)	−0.57 (−2.07–0.93)	−0.12 (−0.65–0.41)
Quartiles of staple food intake					
Q1 (≤4 *liang*/*day*) ^3^	2371	1.00	1.00	Ref.	Ref.
Q2 (5–6 *liang*/*day*)	3450	0.84 (0.79–0.90) **	0.92 (0.86–0.98) **	0.67 (0.46–0.89) **	0.24 (0.04–0.43) *
Q3 (7–8 *liang*/*day*)	1593	0.80 (0.75–0.87) **	0.92 (0.85–0.99) *	0.62 (0.37–0.87) **	0.12 (−0.11–0.34)
Q4 (≥9 *liang*/*day*)	1521	0.79 (0.74–0.85) **	0.91 (0.84–0.98) *	0.82 (0.57–1.07) **	0.26 (0.01–0.50) *

* *p* < 0.05 ** *p* < 0.01; ^1^ CI: confidence interval; ^2^ Models adjusted for age, gender, job before 60 years of age, residence, smoking, alcohol drinking, physical activity (regular exercise), number of chronic diseases, frequency intake of fruit, vegetable. ^3^ 1 *liang (Chinese unit)* equals to 50 g.

**Table 3:** Hazard ratios and differences in median age at death (95% CI) for all-cause mortality according to food frequency intake and other lifestyle factors among Chinese elderly.

	HR (95% CI)		Differences (95% CI) in Median Age at Death (Years)	
	Unadjusted	Multivariable Adjusted ^1^	Unadjusted	Multivariable Adjusted ^1^
Age	1.07 (1.06–1.07) **	1.07 (1.06–1.07) **	−0.19 (−0.21–−0.18) **	−0.18 (−0.19–−0.17) **
Urban *vs.* rural	0.75 (0.72–0.79) **	0.92 (0.87–0.97) **	−0.77 (−0.97–−0.56) **	0.14(0.04−0.32) **
Women *vs.* men	1.07 (1.02–1.12) *	0.79 (0.74–0.84) **	−0.34 (−0.51–−0.17) **	0.66 (0.47–0.85) **
Number of chronic diseases	1.01 (0.98–1.03)	1.06 (1.03–1.09) **	−0.06 (−0.14–0.02)	−0.17 (−0.24–−0.09) **
Manual job *vs.* non-manual job	1.89 (1.69–2.11) **	1.37 (1.21–1.55) **	−2.41 (−2.83–−1.99) **	−0.97 (−1.50–−0.44) **
Smoking status				
Current smokers	1.00	1.00	Ref.^2^	Ref.
Ex-smokers	1.04 (0.95–1.13)	1.00 (0.92–1.10)	−0.21 (−0.51–0.08)	−0.08 (−0.40–0.23)
Non-smokers	1.09 (1.02–1.16) *	0.94 (0.87–1.02)	−0.37 (−0.61–−0.13) **	0.15 (−0.12–0.43)
Non-alcohol drinking *vs.* drinking	0.98 (0.92–1.03)	1.02 (0.96–1.09)	0.06 (−0.15–0.28)	−0.06 (−0.26–0.14)
Having physical activity *vs.* no physical activity	0.60 (0.57–0.64) **	0.73 (0.68–0.77) **	2.05 (1.78–2.31) **	1.18 (0.95–1.41) **
Fruit intake				
Never	1.00	1.00	Ref.	Ref.
Occasionally	0.91 (0.86–0.96) **	0.96 (0.91–1.02)	0.37 (0.17–0.57) **	0.08 (−0.11–0.26)
Daily	0.70 (0.64–0.75) **	0.85 (0.77–0.92) **	1.07 (0.72–1.42) **	0.38 (0.07–0.69) *
Vegetable intake				
Never	1.00	1.00	Ref.	Ref.
Occasionally	0.77 (0.68–0.86) **	0.80 (0.70–0.91) **	0.73 (0.32–1.15) **	0.36 (0.02–0.70) *
Daily	0.63 (0.57–0.70) **	0.74 (0.66–0.83) **	1.41 (1.04–1.79) **	0.60 (0.28–0.91) **
Intake of meat				
Never	1.00	1.00	Ref.	Ref.
Occasionally	1.01 (0.95–1.08)	0.97 (0.90–1.04)	0.05 (−0.18–0.28)	0.17 (−0.06–0.41)
Daily	0.95 (0.88–1.02)	1.05 (0.97–1.14)	0.40 (0.16–0.64)**	0.04 (−0.22–0.30)
Intake of fish				
Never	1.00	1.00	Ref.	Ref.
Occasionally	1.03 (0.98–1.09)	1.06 (1.00–1.13) *	−0.07 (−0.27–0.12)	−0.13 (−0.32–0.07)
Daily	0.86 (0.79–0.93) **	0.94 (0.85–1.03)	0.38 (0.12–0.65) **	0.31 (0.01–0.62) *
Intake of tea				
Never	1.00	1.00	Ref.	Ref.
Occasionally	0.95 (0.89–1.02)	0.98 (0.92–1.05)	0.21 (−0.03–0.45)	0.11 (−0.11–0.32)
Daily	0.80 (0.75–0.85) **	0.95 (0.89–1.02)	0.68 (0.42–0.93) **	0.18 (−0.04–0.41)
Intake of sugar				
Never	1.00	1.00	Ref.	Ref.
Occasionally	1.04 (0.98–1.10)	1.00 (0.95–1.07)	−0.18 (−0.38–0.03)	−0.07 (−0.26–0.13)
Daily	1.05 (0.99–1.13)	1.04 (0.97–1.12)	−0.32 (−0.56–−0.08) **	−0.15 (−0.38–0.08)
Intake of salt-preserved vegetable				
Never	1.00	1.00	Ref.	Ref.
Occasionally	1.02 (0.97–1.08)	1.12 (1.06–1.19) **	−0.05 (−0.26–0.16)	−0.18 (−0.37–0.02)
Daily	0.96 (0.90–1.03)	1.10 (1.03–1.18) **	0.13 (−0.09–0.35)	−0.14 (−0.36–0.07)
Intake of garlic				
Never	1.00	1.00	Ref.	Ref.
Occasionally	0.95 (0.90–1.00) *	0.95 (0.90–1.00)	0.13 (−0.06–0.32)	0.12 (−0.06–0.30)
Daily	0.81 (0.75–0.88) **	0.95 (0.87–1.04)	0.52 (0.21–0.83) **	0.14 (−0.16–0.45)
Intake of egg				
Never	1.00	1.00	Ref.	Ref.
Occasionally	1.06 (0.99–1.13)	1.07 (0.99–1.14)	−0.14 (−0.37–0.09)	−0.16 (−0.39–0.07)
Daily	0.91 (0.85–0.98) *	1.02 (0.94–1.10)	0.25 (0.00–0.49) *	−0.11 (−0.38–0.17)
Intake of beans				
Never	1.00	1.00	Ref.	Ref.
Occasionally	1.00 (0.94–1.07)	1.06 (0.99–1.14)	−0.01 (−0.24–0.22)	−0.17 (−0.40–0.06)
Daily	0.86 (0.80–0.93) **	1.05 (0.96–1.14)	0.48 (0.20–0.76) **	−0.13 (−0.41–0.15)

* *p* < 0.05, ** *p* < 0.01; ^1^ Model adjusted for variables listed in the table. ^2^ Ref. was zero.

In the full multivariable model, compared with non-consumers, daily fruit (HR 0.85 (95% CI 0.77–0.92)) and vegetable intake (HR 0.74 (95% CI 0.66–0.83)) were inversely associated with all-cause mortality (Table 3). In the fully adjusted model, the amount of alcohol was not associated with mortality (data not shown). The associations remained after excluding those died within one year of follow up.

In univariate analysis, there was an inverse association between daily bean intake and mortality. However, this inverse association turned to a positive association after adjusting for age and gender. In a parsimonious model (adjusted for the same covariates as the multivariable model in Table 2), the HR (95% CI) for mortality were: 1.11 (1.04–1.18) for occasional bean intake and 1.07 (0.99–1.15) for daily bean intake. Comparing bean consumers with non-consumers, the HR for mortality was 1.10 (1.03–1.17) (*p* = 0.004).

**Figure 1:**
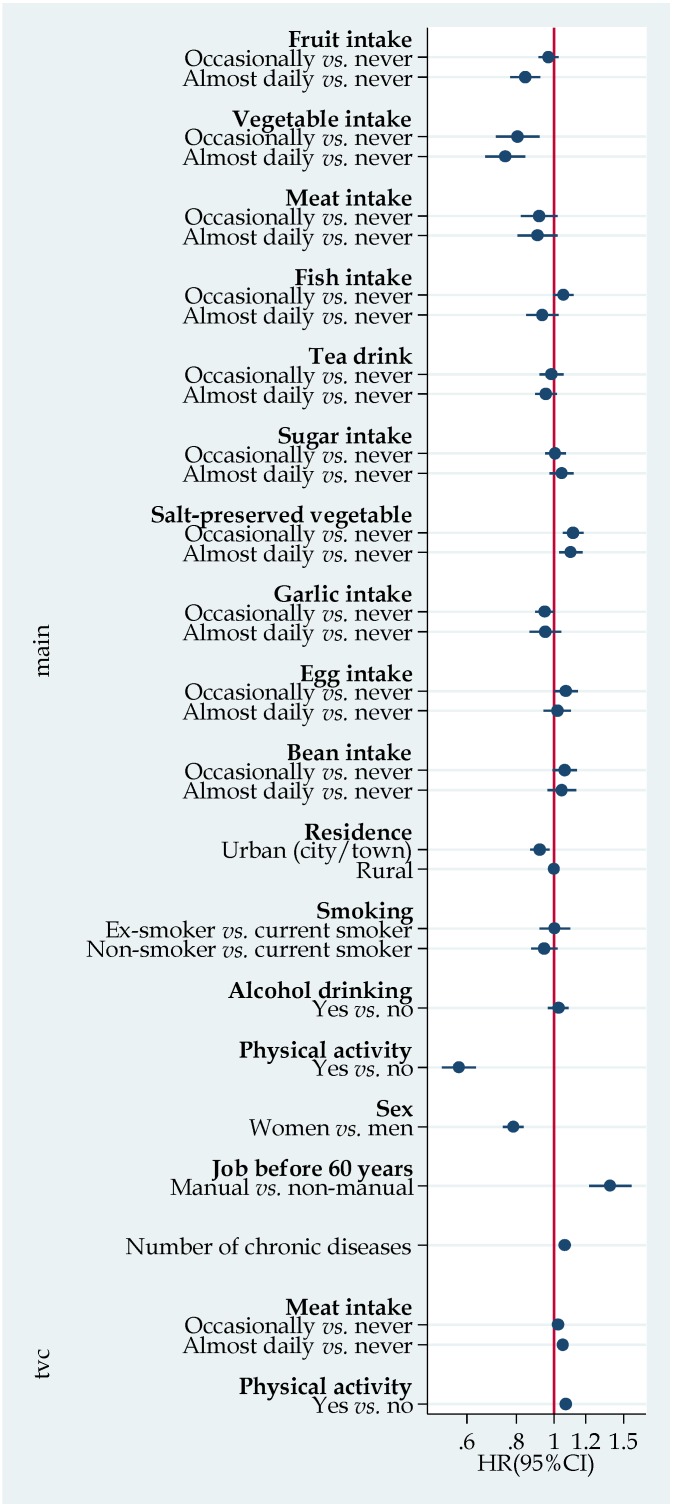
Hazard ratio (HR) (95% CI (Confidence Interval)) for all-cause mortality according to lifestyle factors. Model adjusted for age, and all the variables listed in the figure. The time-varying covariate (TVC) section provided results on the interaction between meat intake, physical activity and time (years). TVC represents covariate × time interaction. HRs for daily meat × time and physical activity × time was 1.05 (95% CI 1.02–1.08) and 1.07 (95% CI 1.05–1.09), respectively.

Figure 1 shows the results of Cox regression with time-varying interaction with meat and physical activity. Meat frequency intake was inversely associated with mortality. However, the effect decreased with time. When we further stratified the analyses by chronic disease status, most of the associations were similar between those with or without chronic disease (Figure S1). However, an inverse association between non-smoking and mortality was only seen among those with chronic diseases. A positive association between alcohol drinking and mortality was found among those with chronic disease (HR 1.11 95% CI 1.01–1.21) but not those without chronic disease.

Kaplan-Meier survival curve shows a clear difference in the proportion of survival according to vegetable intake (Figure S2). In the fully adjusted Laplace regression models, median age at death for participants who ate vegetables daily was about 0.6 years older than those who did not eat vegetables (difference in median age at death 0.60 (95% CI 0.28–0.91) years. Participants who consumed fruit daily survived a median of 0.39 (95% CI 0.07–0.69) years longer than those who never ate fruit.

In general, results from the Cox regression and the Laplace regression are similar. Discrepancies were seen for salt-preserved vegetable and fish intake with the two different methods. However, the directions of the associations in the two models were the same. Daily intake of fish seemed to be beneficial for survival in the Laplace regression model. Consumption of salt-preserved vegetables was associated with increased risk of mortality.

**Figure 2:**
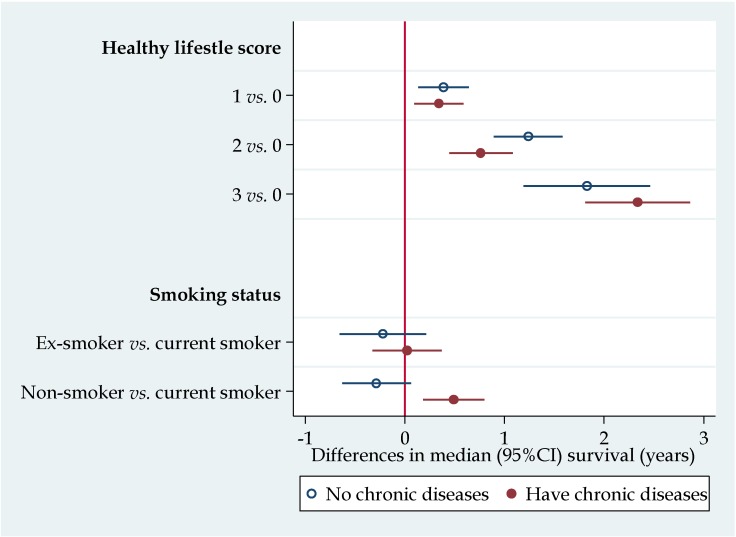
Median differences of survival according to healthy lifestyle score and smoking status stratified by status of chronic diseases. Results were from Laplace regression, adjusted for age, sex, residence, job (manual *vs.* non-manual) before age of 60 years, and alcohol drinking. Healthy lifestyle score included daily intake of fruit, vegetable, and having physical activity. Each factor scored 1. CI: Confidence Interval.

Figure 2 shows the median differences in survival by the healthy lifestyle score. There was a dose response relationship between the healthy lifestyle score and survival. Compared with those who had a lifestyle score of zero, those who scored three had two years longer median survival. Among those with one or more chronic diseases, smoking was associated with 0.5 years shorter survival than non-smoking. Excluding those with follow-up less than one year or having ADL disability did not change the main findings (data not shown). Among those with chronic conditions, smokers had shorter median survival as compared with non-smokers.

## 4. Discussion

In this first cohort study on nutrition, lifestyle and mortality in China focusing on the oldest old, we found food habits are related to mortality risk. While fruit and fresh vegetable intake were inversely associated with mortality risk, salt preserved vegetable intake was associated with increased risk of mortality. High frequency intake of protein rich food including meat, fish, bean and egg was not protective against mortality risk.

Consistent with previous studies [25], we found that women had lower mortality risk than men, and that undertaking physical activity, and having a non-manual job before 60 years of age were positively associated with survival. Non-smokers had a reduced risk of mortality compared to smokers. However, the HRs for mortality were similar between current and ex-smokers. As we did not have information of age of quitting smoking, we were not able to further investigate this association.

The beneficial effects of fruit and vegetable are well known. In a recent systematic review, intake of fruit and vegetable was found to be associated with reduced risk of mortality [15]. Our findings extend the evidence of the beneficial effects of fruit and vegetable intake to the oldest old.

In the general population, high protein intake has been found to be associated with increased risk of mortality [19,23,24]. It is commonly believed that the elderly need more protein. The prevalence of inadequate intake of protein is high in Chinese elderly [22]. However, in this study we found that high frequency intake of protein rich food including fish, bean and eggs were associated with increased mortality. A high intake of egg may be associated with cholesterol intake and thus diabetes [31,32]. Discrepancies were found between the Cox regression and the Laplace regression in the association between fish intake and mortality.

Staple food intake was not related to mortality. The mean intake of staple food was around 6 *liang* (300 g/day). This intake is much higher than that of Western countries. Neither high intake of rice nor wheat flour is related to the risk of mortality in our study. This result is in line with findings from two large Japanese studies [17,18] and a regional study in China [33]. Rice intake may increase the risk of diabetes, although it does not increase the risk of cardiovascular disease (CVD) mortality. High dietary glycemic index was inversely related to mortality in men [34]. However, due to the lack of information of energy intake, caution needs to be taken when interpreting our results.

In China, there is an unwavering belief in the benefits of vegetables and tofu (as shown by the old Chinese saying “*vegetables and tofu keep you healthy*”). Consequently, it is not surprising that as many as 80% of the elderly ate beans. What was surprising however was our finding of an increased risk of mortality among bean eaters as compared with non-bean eaters. It could be that elderly with chronic disease may have changed their dietary habits by increasing bean intake. However, even adjusting for the number of chronic diseases the above association still existed. Whether the way the beans were grown and cooked could explain the link remains to be studied. In the study, we are not able to separate the intake of different types of beans/bean products.

We found that the intake of salt-preserved vegetables (occasionally or daily) was associated with about 10% increased risk of all-cause mortality. This finding is consistent with current knowledge. Salt-preserved vegetables have high levels of sodium. It is known that high sodium intake increases the risk of hypertension and CVD. Pickled vegetable intake has previously been shown to increase the risk of cancer [35]. In this study, about 20% of the elderly reported that they had a daily intake of salt-preserved vegetables. However, it is not valid to conclude that as one in five of the oldest old consumed salt-preserved vegetables, it should be a component of healthy lifestyle as suggested by one study in China [36]. Such a claim is misleading and harmful. Limiting salt-preserved vegetable intake and replacing with fresh or other forms of preserved vegetables should be encouraged.

Our study suggested the additive benefits of healthy lifestyle factors. Having three healthy lifestyle factors (daily intake of fruit, vegetable and having physical activity) was associated with a two-year increase in survival among this oldest old cohort. This finding is consistent with previous studies on healthy lifestyle and mortality among elderly in developed countries [20,25]. It provides strong evidence for promoting fruit and vegetable consumption as well as physical activity among the elderly.

The study has several limitations. Firstly, we only have self-reported information on frequency intake of most of the food items. No detailed dietary intake information was collected including the amount of fruit, vegetables and other foods. Secondly, the death information was based on interviews with family members. No death record review by doctors was undertaken. We thus did not further investigate the association between food habits and cause-specific mortality. Thirdly, as those who had a poorer diet or lifestyle in their middle years may have died before the inception of the cohort, selective healthier survivor biases are likely in studies conducted only in very old subjects. About 25% of the cohort has been lost to follow-up. However, most of the lost to follow-up were due to city construction and moving of living places. This may not generate substantial bias. Data was not available on height; thus, body mass index (BMI) could not be calculated and only weight was modeled as a potential confounder. Chronic diseases were self-reported. It may explain why among such an older cohort the mean number of chronic diseases was less than one. Therefore, after adjustment for chronic diseases, some residual confounding may still persist in the association between food intake (which is obviously related to disease patterns) and mortality. Finally, as the study population is mostly non-obese, the associations identified in the study may be different for overweight and obese older people. The strength of the study is the large sample size and long duration of follow-up. The findings can be generalized to the elderly population in China.

## 5. Conclusions

Diet and lifestyles are related to mortality among Chinese people aged 80 years and above. Frequency intake of fruit and vegetable is inversely, but salted vegetable is positively associated with all-cause mortality. Undertaking physical activity is beneficial for the prevention of pre-mature death. Future studies on overall dietary patterns based on multiple 24-hour food records and mortality are warranted. While promoting the consumption of vegetable for health, limiting the consumption of salted vegetable should be emphasized.

## Supplementary Files

Supplementary File 1
