# Supplementary material for: Food Habits, Lifestyle Factors and Mortality among Oldest Old Chinese: The Chinese Longitudinal Healthy Longevity Survey (CLHLS)
Source: Nutrients. 2015 Sep 9;7(9):7562–79. doi: 10.3390/nu7095353 (PMC4586548; doi:10.3390/nu7095353)
Supplement: Supplementary File 1 [file nutrients-07-05353-s001.docx]

Supplementary Materials

^^

**Figure S1.** Hazard ratio (95% confidence interval (CI)) for all-cause mortality according to lifestyle factors stratified by chronic disease status. Model adjusted for age, and all the variables listed in the figure. The time-varying covariate (TVC) section provided results on the interaction between meat intake, physical activity and time (years). TVC represents covariate x time interaction.

**Figure S2.** Kaplan-Meier survival curve by intake of vegetable

**Table S1.** Baseline characteristics of participants with and without information on mortality.

|  | **With mortality information** | **Without mortality information** | ***p*-value** |
| --- | --- | --- | --- |
| *N* | 6,753 | 2,206 |  |
| Age, mean (S.D.) | 93.2 (7.5) | 89.5 (7.3) | <0.001 |
| Sex |  |  | 0.006 |
| Men | 2,634 (39.0%) | 933 (42.3%) |  |
| Women | 4,119 (61.0%) | 1,273 (57.7%) |  |
| Years of education |  |  | <0.001 |
| No | 4,721 (69.9%) | 1,281 (58.1%) |  |
| 1–5 | 1,402 (20.8%) | 479 (21.7%) |  |
| 6–9 | 377 (5.6%) | 191 (8.7%) |  |
| >9 | 219 (3.2%) | 239 (10.8%) |  |
| Missing | 34 (0.5%) | 16 (0.7%) |  |
| Co-residence |  |  | <0.001 |
| With household member(s) | 5,816 (86.1%) | 1,814 (82.2%) |  |
| Alone | 635 (9.4%) | 264 (12.0%) |  |
| Nursing home | 301 (4.5%) | 128 (5.8%) |  |
| Missing | 1 (<1%) | 0 (0.0%) |  |
| Residence |  |  | <0.001 |
| Urban | 2,209 (32.7%) | 1,169 (53.0%) |  |
| Rural | 4,544 (67.3%) | 1,037 (47.0%) |  |
| Smoking status |  |  | <0.001 |
| Current smoker | 1,150 (17.0%) | 363 (16.5%) |  |
| Ex-smoker | 976 (14.5%) | 397 (18.0%) |  |

**Table S1.** *Cont.*

|  | **With mortality information** | **Without mortality information** | ***p*-value** |
| --- | --- | --- | --- |
| Non-smoker | 4,623 (68.5%) | 1,444 (65.5%) |  |
| Alcohol consumption |  |  | 0.001 |
| Yes | 1,643 (24.4%) | 462 (21.0%) |  |
| Not at present | 5,102 (75.6%) | 1,742 (79.0%) |  |
| Exercise |  |  | <0.001 |
| No physical activity at present | 5,199 (77.0%) | 1,345 (61.1%) |  |
| Undertaking physical activity | 1,549 (23.0%) | 857 (38.9%) |  |
| Quartiles of other activities score |  |  | <0.001 |
| Q1 (0) | 2,065 (30.6%) | 370 (16.8%) |  |
| Q2 (3–7) | 2,113 (31.3%) | 561 (25.4%) |  |
| Q3 (9–14) | 1,372 (20.3%) | 584 (26.5%) |  |
| Q4 (15–56) | 1,203 (17.8%) | 691 (31.3%) |  |
| ADL disability |  |  | <0.001 |
| No | 4,071 (60.3%) | 1,528 (69.3%) |  |
| Yes | 2,658 (39.4%) | 664 (30.1%) |  |
| Missing | 24 (0.4%) | 14 (0.6%) |  |
| Number of chronic diseases, mean (SD) | 0.8 (1.0) | 0.9 (1.2) | <0.001 |
| Intake of fruit |  |  | <0.001 |
| Never | 2,049 (30.4%) | 536 (24.3%) |  |
| Occasionally | 3,742 (55.5%) | 1,090 (49.5%) |  |
| Almost daily | 953 (14.1%) | 578 (26.2%) |  |
| Intake of vegetable |  |  | 0.002 |
| Never | 351 (5.2%) | 75 (3.4%) |  |
| Occasionally | 1,219 (18.1%) | 397 (18.0%) |  |
| Almost daily | 5,175 (76.7%) | 1,732 (78.6%) |  |
| Intake of meat |  |  | <0.001 |
| Never | 1,264 (18.9%) | 480 (21.9%) |  |
| Occasionally | 3,471 (51.8%) | 977 (44.6%) |  |
| Almost daily | 1,970 (29.4%) | 733 (33.5%) |  |
| Intake of fish |  |  | <0.001 |
| Never | 2,116 (31.7%) | 701 (32.2%) |  |
| Occasionally | 3,791 (56.7%) | 1,124 (51.6%) |  |
| Almost daily | 777 (11.6%) | 352 (16.2%) |  |
| Intake of tea |  |  | <0.001 |
| Never | 3,786 (58.5%) | 1,073 (51.0%) |  |
| Occasionally | 1,132 (17.5%) | 365 (17.4%) |  |
| Almost daily | 1,552 (24.0%) | 664 (31.6%) |  |
| Intake of sugar |  |  | <0.001 |
| Never | 2,059 (30.7%) | 734 (33.6%) |  |
| Occasionally | 2,901 (43.3%) | 846 (38.8%) |  |
| Almost daily | 1,738 (25.9%) | 603 (27.6%) |  |
| Intake of salt-preserved vegetables |  |  | 0.075 |
| Never | 2,969 (44.6%) | 1,001 (46.0%) |  |
| Occasionally | 2,256 (33.9%) | 680 (31.3%) |  |

**Table S1.** *Cont.*

|  | **With mortality information** | **Without mortality information** | ***p*-value** |
| --- | --- | --- | --- |
| Almost daily | 1,434 (21.5%) | 494 (22.7%) |  |
| Intake of garlic |  |  | 0.001 |
| Never | 3,219 (48.8%) | 1,104 (51.5%) |  |
| Occasionally | 2,688 (40.7%) | 783 (36.5%) |  |
| Almost daily | 690 (10.5%) | 257 (12.0%) |  |
| Intake of egg |  |  | <0.001 |
| Never | 1,399 (20.9%) | 405 (18.5%) |  |
| Occasionally | 3,473 (51.8%) | 957 (43.8%) |  |
| Almost daily | 1,835 (27.4%) | 823 (37.7%) |  |
| Intake of beans |  |  | <0.001 |
| Never | 1,266 (18.8%) | 412 (18.8%) |  |
| Occasionally | 3,998 (59.5%) | 1,156 (52.7%) |  |
| Almost daily | 1,458 (21.7%) | 625 (28.5%) |  |
| Staple food (*liang*/d), mean (SD) | 6.2 (2.5) | 6.0 (2.5) | 0.034 |

**Table S2.** Association between levels of activities other than regular exercise and all-cause mortality^1^.

|  | **HR(95% CI)** |
| --- | --- |
| Quartiles of other activities^2^ |  |
| Q1 | 1.00 |
| Q2 | 0.68 (0.62–0.75)** |
| Q3 | 0.54 (0.48–0.61)** |
| Q4 | 0.36 (0.32–0.42)** |
| Q2 × time | 1.06 (1.03–1.09)** |
| Q3 × time | 1.07 (1.04–1.11)** |
| Q4 × time | 1.13 (1.10–1.17)** |

** *p* < 0.01; ^1^ Model adjusted for age, gender, frequency intake of fruit, vegetables, salted vegetables, smoking, drinking alcohol, physical activity (regular exercise), residence, number of chronic diseases. Cox model with time-varying covariates (physical activity, other activities) were used to assess the association. The inverse association between other activity and all-cause mortality decreased over time. For example, the HR at five years was 0.36 × 1.13^5^ = 0.67 for Q4 as compared with Q1.; ^2^ Including housework, grow vegetables/other field work, garden work, read newspapers/books, raise domestic animals, play cards/or mah-jongg, watching TV and/or listening to the radio, religious activities. CI: confidence interval.

© 2015 by the authors; licensee MDPI, Basel, Switzerland. This article is an open access article distributed under the terms and conditions of the Creative Commons Attribution license (http://creativecommons.org/licenses/by/4.0/).
